# Tracking global development assistance for trauma care: A call for advocacy and action

**DOI:** 10.7189/jogh.11.04007

**Published:** 2021-03-27

**Authors:** Sara M Hollis, Stas Salerno Amato, Eileen Bulger, Charles Mock, Teri Reynolds, Barclay T Stewart

**Affiliations:** 1 World Health Organization, Geneva, Switzerland; 2 Department of Surgery, University of Vermont Medical Center, Burlington, Vermont, USA; 3 Department of Surgery, University of Washington, Seattle, Washington, USA; 4 Department Global Health, University of Washington, Seattle, Washington, USA; 5 Harborview Injury Prevention and Research Center, Seattle, W Washington A, USA

## Abstract

**Background:**

This study aimed to track development assistance for trauma care (DAH-TC), uncover funding trends and gaps, and compare DAH-TC to development assistance for other health conditions.

**Methods:**

A systematic search of the OECD Creditor Reporting System (CRS) and Development Assistance Committee (DAC) databases was performed to capture projects related to trauma care. Reports from large foundations and public-private partnerships were also searched. DAH-TC was described, and comparisons were made between DAH-TC and other health conditions.

**Results:**

The search yielded 1754 records; after applying exclusion criteria, 301 records were included for analysis. During the 25-year period, US$93.7M of DAH-TC was disbursed to low- and middle-income countries (LMICs) (0.02% of total DAH). Contributions were dominated by a few donors and fluctuated dramatically over time. A sizable portion of DAH-TC came in the form of investments to build infrastructure (38% of DAH-TC); information and research activities (17%); and training (16%). Nearly US$58M (62% of DAH-TC) was funneled to projects that targeted victims of war. Trauma care received US$0.04 per DALY incurred, while malaria, TB, HIV and MCH received US$9.62 per DALY, US$25.09 per DALY, US$4.05 per DALY and US$45.75 per DALY, respectively.

**Conclusions:**

DAH-TC is critically underfunded, particularly compared to other health foci. To improve the DAH-TC landscape, stakeholders can better mobilize domestic resources; use advocacy more effectively by catalyzing network convergence, grafting trauma care onto related high-priority issues, and seeking broader coalitions; and develop partners within the donor and channel communities to promote strategic DAH-TC disbursements.

Nearly five million people die from injury each year, and tens of millions more are left with disability; nearly 90% of this burden occurs in low- and middle-income countries (LMICs) [1]. Despite the enormous economic, social, and health burdens incurred by injury, many LMIC health care systems remain ill-prepared to provide timely trauma care due to critical deficiencies in political will, funding, organization, and resources [2]. This is caused, in part, by appropriation of resources to more high-profile health conditions that incur markedly smaller burdens of disease than injury (eg, HIV, tuberculosis, malaria) [3]. Opportunities to prevent injury occur through a range of environmental, educational and legislative pathways and have proved effective in saving lives [4]. However, prevention mechanisms alone will not solve the burden; an organized trauma care system is essential to address injuries if and when they occur, Given that well-planned trauma care can avert preventable death and disability, LMICs currently without the financial capacity to strengthen their trauma care systems urgently need targeted development assistance.

Development assistance represents financial and in-kind contributions by donors (eg, high-income countries, foundations, public-private partnerships, philanthropy) to recipient LMICs [5]. Monies given for health sector initiatives are termed development assistance for health (DAH). Intermediary channels are staffed with experts to ensure that donations are used for meaningful programming and typically disburse DAH. These channels also uncouple donations from donor geopolitical strategy, provide a secondary layer of accountability, and distribute money over time, which mitigates funding cycles and donor volatility.

Much is known about the DAH landscape in general, and for high profile global health conditions (eg, maternal and child health, HIV, tuberculosis, malaria) [6]. However, the funding landscape for trauma care is unknown [7]. Understanding the DAH for trauma care (DAH-TC) can promote financial accountability and facilitate more effective advocacy for funding of interventions that reduce the burden of injury. Making transparent the DAH-TC is not only necessary for informed short-term decision-making at the global and national levels but may have downstream effects on long-term health policy decisions and investments in sustainable trauma care programs.

To address the lack of DAH-TC landscape data and promote transparency around global health funding, this study aimed to track DAH-TC, uncover funding trends and shortfalls, and compare DAH-TC to development assistance for other high-profile global health conditions, both in absolute terms and relative to disease burden. We hypothesized that DAH-TC will have a vulnerable financial landscape (eg, few donors and channels) and have markedly less DAH compared to other high-profile global health conditions, both in absolute terms and relative to disease burden.

## MATERIALS AND METHODS

### Data acquisition

A systematic search of the Institute of Health Metrics and Evaluation (IHME) Financing for Global Health 2015 DAH Database (IHME DAH Database), which draws from the Creditor Reporting System (CRS) and Development Assistance Committee (DAC) databases of the Organization for Economic Cooperation and Development (OECD), was performed to capture all OECD-reported projects related to trauma care, and their corresponding financial disbursements from 1990-2015 (most recent database update) [5]. Project titles and descriptions were queried to extract all project-level entries from the IHME DAH database where keywords relating to trauma care were present. The keyword list (see Appendix S1 in the **Online Supplementary Document**) was generated from terms within: WHO injury products (eg, Guidelines for Essential Trauma Care [8], Emergency Care Systems Framework and Health Systems Building Blocks [9], Global Initiative for Emergency and Essential Surgical Care [10]); existing IHME groupings and phylogeny [11]; and ICD-10 injury codes (S00-T88) [12]. The IHME DAH Database was the preferred source for obtaining DAC and CRS project records vs accessing raw data from OECD repositories directly. This is due to the rigorous econometric corrections applied by IHME to account and correct for underreporting, commitments not actually disbursed and double-counting due to multiple transfer points between channels.(Institute for Health Metrics and Evaluation, 2016a) Disbursements from other funding entities (eg, Gavi, the Vaccine Alliance (Gavi), the Global Fund to Fight Aids, Tuberculosis and Malaria (GFATM), The Bill and Melinda Gates Foundation (BMGF), Development Banks and UN Agencies) are only included in OECD Databases when acting as intermediary channels funded directly by OECD donor governments – which has historically been ameliorated by obtaining disbursement data through personal correspondence and screening financial statements, online grants databases and annual reports, as documented in the IHME Methods Annex [11]. In keeping with this methodology, we replicated this approach to the extent that we had access by conducting a grey literature search of annual reports and open-source disbursement information to reveal any additional funding sources for trauma care during this period. The Agencies, foundations and NGOs of interest (see Appendix 2 in the **Online Supplementary Document**) were selected based on which demonstrated the most measurable funding in their respective categories in the *Financing Global Health 2015* Report [13].

All records retrieved by the keyword search underwent a fully-crossed review by two independent coders (SH, SA) to target only projects related to trauma care and to exclude irrelevant records – namely, projects focusing on non-trauma emergency care, system-wide approaches, psychosocial trauma or injury prevention. Although the importance of injury prevention is recognized, it was excluded here as the related development assistance involves a number of non-health sectors with established DAH funding streams that are only partially represented in health funding databases. Records of DAH-TC before 1990 were excluded since the CRS was not well established until that year, as were projects with an unclear description. For any non-English entries, Google Translate (Google Inc., USA) was used to translate project descriptions. Any discrepancies were resolved by a third reviewer (BS), and the overall inter-rater agreement was less than 5%.

### Data analysis

Using project descriptions, identified trauma care projects were categorized into various themes (eg, infrastructure development, information technology and research, training and human resources) and sites where the intervention occurred along the trauma system (eg, World Health Organization Emergency Care System Framework components such as pre-hospital care, facility-based care, surgical care, rehabilitation; see Appendix 3 in the **Online Supplementary Document**) [9]. DAH-TC was then mapped according to donors, channels, and recipients in accordance with IHME guidelines. Lastly, descriptive comparisons were made between DAH-TC and other high priority global health conditions using publicly available data on DAH and disease burdens from IHME [1].

## RESULTS

**Figure 1:**
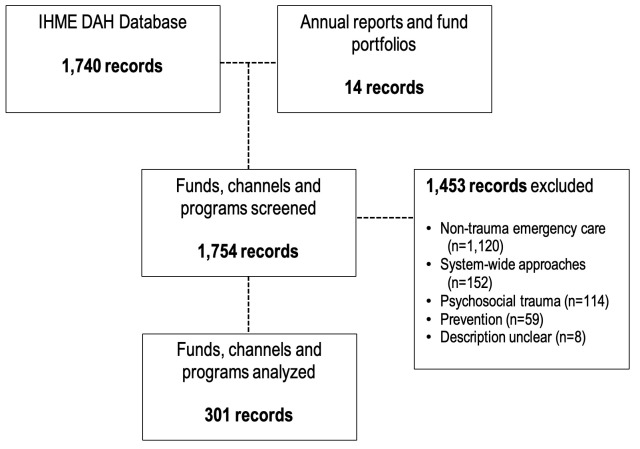
Flow diagram for systematic search trauma-related disbursements.

**Figure 2:**
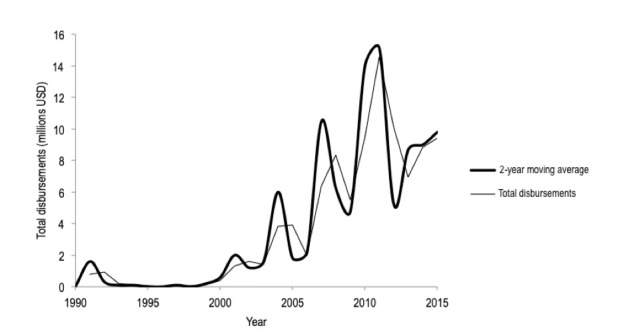
Total disbursements in the global development assistance for trauma care (DAH-TC) from 1990- 2015. USD – United States Dollars.

**Figure 3:**
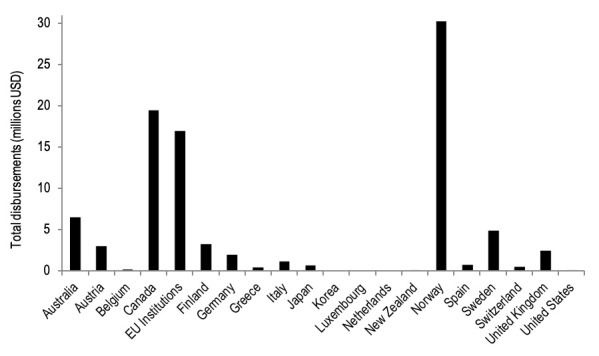
Donors in the global development assistance for trauma care (DAH-TC) landscape from 1990- 2015. USD – United States Dollars, EU – European Union.

The search yielded 1754 records (ie, donations, channel disbursements, recipient funding) related to trauma care from 1990 to 2015. After screening, only 301 records were related to trauma care (**Figure 1**). During the 25-year period, US$93.7M of DAH-TC was disbursed to LMICs (**Figure 2**), representing 0.02% of total DAH funding during that period. Nineteen high-income countries, in addition to the European Commission (EC), contributed to DAH-TC for LMICs (**Figure 3**). Contributions were dominated by a few donors, namely Norway (US$30M), Canada (US$19M) and the European Commision (US$17M), and fluctuated dramatically over time. Notably, common large DAH donors did not give monies to trauma care (eg, United States, The Bill and Melinda Gates Foundation, United Kingdom, Germany, France, Japan).

**Figure 4:**
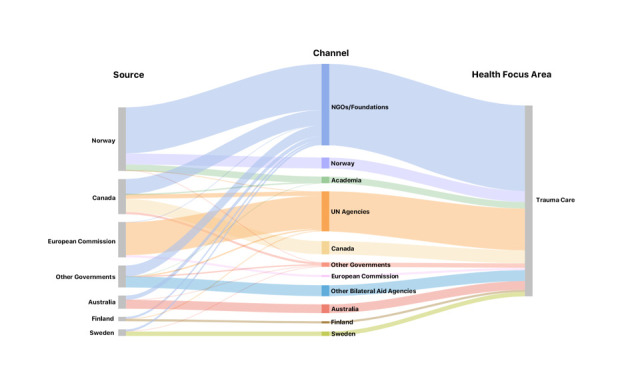
Financing flow depicting the donors and channels for global development assistance for trauma care (DAH-TC) from 1990- 2015. NGO – non-governmental organization, Univ. – university, UN – United Nations.

Seventy percent of DAH-TC flowed through a channel (**Figure 4**); the largest channels were non-governmental organizations (NGOs), which dispersed US$37.2M (40% of DAH-TC), and UN agencies, which disbursed US$19.3M (21%). Unlike the funding landscapes for other health foci, the lack of well-established channels for DAH-TC has allowed academia and bilateral partnerships to become visible intermediaries.

**Figure 5:**
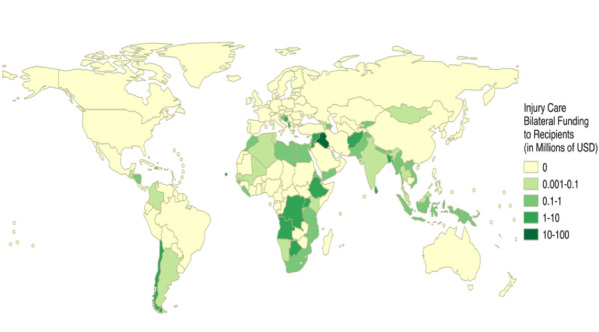
Global development assistance for trauma care (DAH-TC) recipients from 1990-2015. USD – United States Dollars, Recipients listed in descending value of DAH-TC: Iraq 18.13, Albania 6.23, Lebanon 5.68, Afghanistan 4.10, Cambodia 3.96, Syrian Arab Republic 3.43, Haiti 3.21, China (People's Republic of) 2.83, Bangladesh 2.66, Uganda 2.27, Angola 2.15, Ethiopia 2.07, Botswana 1.89, Sri Lanka 1.66, Bosnia and Herzegovina 1.64, Democratic Republic of the Congo 1.29, Jordan 1.17, Viet Nam 0.98, South Africa 0.96, Croatia 0.83, Pakistan 0.76, Sierra Leone 0.68, Egypt 0.64, Lao People's Democratic Republic 0.59, Libya 0.54, Yemen 0.52, Papua New Guinea 0.52, Tanzania 0.48, Nicaragua 0.43, Morocco 0.41, Malawi 0.39, Nepal 0.34, Mozambique 0.33, Guinea-Bissau 0.30, Honduras 0.29, Mali 0.28, Liberia 0.24, Kyrgyzstan 0.20, Eritrea 0.19, Burundi 0.17, Indonesia 0.15, Myanmar 0.14, Bhutan 0.14, Rwanda 0.10, Azerbaijan 0.10, India 0.10, Georgia 0.09, Namibia 0.09, Mongolia 0.08, Colombia 0.07, Thailand 0.05, Algeria 0.05, Kenya 0.05, Guatemala 0.03, Senegal 0.01, Tunisia 0.01.

**Figure 6:**
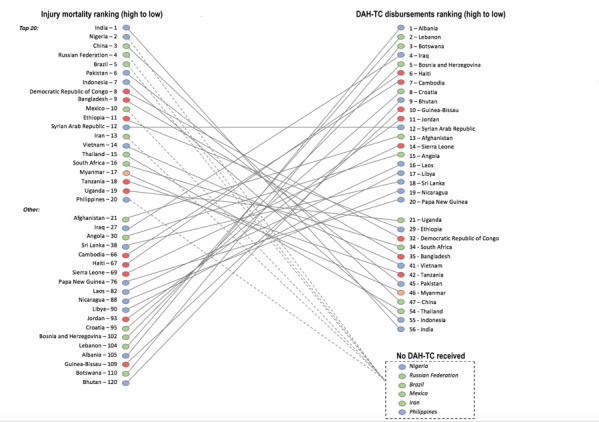
Comparison between 2013 injury mortality rates and development assistance for trauma care (DAH-TC) disbursements from 2000-2015. Injury mortality and development assistance for trauma care (DAH-TC) rankings are 2012 population adjusted; green – high-income countries; blue – upper-middle income countries; orange – lower-middle income countries; red – low-income countries.

Fifty-seven countries received DAH-TC (**Figure 5**). The Eastern Mediterranean Region dwarfed the dollar amount received by any other region at US$37M (40% of DAH-TC); the highest country recipient was Iraq (US$18.1M, 19.3% of DAH-TC), which received over thirteen times the average dollar amount received by all recipients and US$0.55 per capita. Other top recipients include Albania, Lebanon, Afghanistan, Cambodia, and Syria. Typical recipients of DAH for other health foci were conspicuously absent, such as countries in sub-Saharan Africa, Latin America, South Asia, and Southeast Asia. **Figure 6** demonstrates that many countries with the highest population-adjusted injury mortality rates receive far fewer population-adjusted DAH-TC disbursements than countries with lower disease burdens, representing a gross mismatch in the appropriation of available DAH-TC.

The most sizable portion of DAH-TC (38% of DAH-TC) came in the form of investments to build infrastructure (eg, facilities, equipment, transport, communication). The next largest target categories were information and research (eg, injury surveillance activities, development and maintenance of trauma registries) followed by human resources and training (eg, lay responders, pre-hospital providers, health care professionals) at 17% and 16% of total DAH-TC, respectively. Nearly US$58M (62% of DAH-TC) was funneled to projects that targeted victims of war. Notably, war incurs only 3% of the global injury burden.

**Figure 7:**
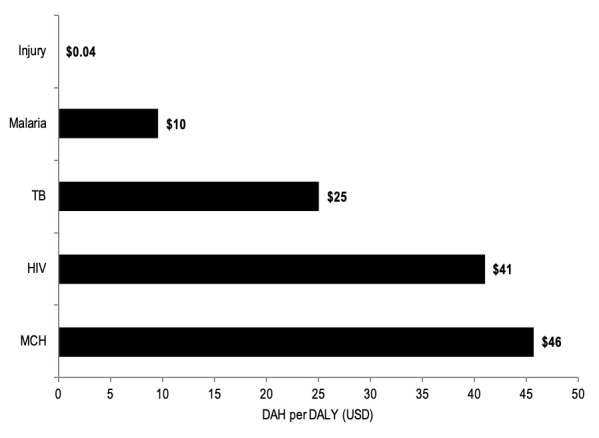
Median global development assistance for trauma care (DAH-TC) per disability-adjusted life year (DALY). USD – United States Dollars.

DAH-TC constitutes only a small fraction of total DAH (**Figure 7**). For context, trauma care received 0.05% of the DAH that was disbursed to HIV, TB, malaria, and maternal and child health. When comparing 4-year median aggregate DAH from 2000 to 2013 based on 2013 disability-adjusted life year (DALY) estimates, trauma care received US$0.04 per DALY incurred, while malaria, TB, HIV and MCH received US$9.62 per DALY, US$25.09 per DALY, US$4.05 per DALY and US$45.75 per DALY, respectively.

## DISCUSSION

This study aimed to track DAH-TC, elucidate funding trends and gaps, and compare DAH-TC to DAH for other high-profile global health conditions, both in absolute terms and relative to disease burden. There are several main findings. First, development assistance for trauma care (DAH-TC) is critically underfunded, and far from commensurate to the disease burden attributable to injury, particularly when compared to other high-profile global health foci. Second, the donor and channel landscapes are sparse, creating vulnerability to temporal economic trends (eg, recessions) or shifts in donor priorities. Third, current DAH-TC appears tied to geopolitical priorities, instead of being driven by the health burdens of populations in need and the limited resources currently available to address them in LMICs.

Although DAH-TC increased nearly 900% between 1990 and 2015, this generally followed the overall trend in DAH, and remains grossly inadequate relative to the anticipated costs of trauma care capacity improvements needed in LMICs [14]. Further, increases in DAH-TC were dwarfed by DAH increases for other health foci over the same time period (eg, TB 4850%; malaria 4051%; HIV/AIDS 2964%) whose respective and combined burdens of disease are substantially less than that of injury [1,15]. DAH-TC dispersed per DALY incurred by injury is only USD 0.04; when compared to that for maternal and child health (US$46 per DALY incurred by maternal and child health conditions) or HIV (US$41 per DALY incurred by HIV), the critical funding gap for injury becomes immediately apparent. Making up this gap will require mobilization of domestic resources, ensuring financial accountability, and advocating for DAH-TC increases at the donor-level and requests at the recipient-level.

Domestic financing has increased in many countries with high burdens of injury, and in many cases, is no longer inconsequential compared to DAH [16]. Therefore, much might be gained from mobilization of domestic resources for trauma care, which may also have positive effects beyond improving care for the injured [17]. First, it allows governments to deliver an essential public good to their citizens, thereby strengthening national ownership of this health focus; second, by systematically delivering trauma care, the government can create a contract between it and its citizens helping to legitimize and promote its longitudinal development; third, there is overwhelming evidence that curbing injury burdens can boost national income growth and welcome large welfare gains; and fourth, domestic resource mobilization is a facet of the broader good governance for health agenda that is required to catalyze greater progress toward universal health coverage and attainment of the Sustainable Development Agenda [18-20].

Successful examples of domestic resource mobilization for health sector improvements exist. El Salvador raised tax revenues a total of 4% of GDP between 2005 and 2013 and used that increased revenue to cut child mortality by 75% through sustainable system-wide improvements [21]. Similarly, Rwanda aimed to resurrect and rebuild its health sector after four years of civil war and genocide [22]. The Rwandan government increased tax revenue as a percent of GDP from 3.6% in 1994 to 13.4% in 2013; expenditures were then ‘reoriented’ toward essential health services, markedly lowering mortality for a number of conditions and establishing a culture of national ownership of health system strengthening initiatives [23,24]. Further, investment in the critical platforms for trauma care delivery (ie, pre-hospital system, emergency care, radiology and laboratory services, surgical care, rehabilitation) are the substrate for improving outcomes from a range of conditions, and represent core health systems strengthening targets [3,25].

However, additional domestic resources will continue to be unavailable for some LMICs and inadequate for many others. Therefore, dramatic increases in DAH-TC will be necessary to meet global demand and will require substantial advocacy efforts to alter the funding portfolios of donors. As is often the case when dealing with overlooked issues, advocacy and politics will become all the more important in crafting an improved agenda. Brief examination into the ways in which maternal and child mortality became a global health priority, through the lens of Schiffman and Smith’s policy framework, is useful for considering approaches to advocate for improving trauma care [26]. Early attention to the problem of maternal and child mortality by global health and development technocrats laid a facilitative framework for advocacy network convergence and priority generation at the donor-level [27]. Simultaneously, women’s rights activists established maternal and child survival as a social justice issue and grafted the cause onto a rapidly expanding global norm. Doing so granted the issue status on the global health and development policy agendas and was the foundation from which the maternal survival advocacy network emerged to advance the issue and realize funding needs. Lastly, policy regarding maternal and child health expanded with coalition building, particularly when the issue was framed beyond the health sector. For example, maternal survival benefited from widely resonating social justice and gender equity framing, which generated a broader political coalition. In short, a global health priority is more likely to emerge from a confluence of factors, rather than any single cause; and the creation of a priority may depend on the creation of a broader political coalition to realize the “power of actors” that extends beyond the largely technically oriented stakeholders who may first bring attention to a problem [26,27]. In the case of trauma care, health care providers should engage in advocacy efforts via professional societies is an area that has been underutilized in the past but holds great promise in identifying local and urgent needs on behalf of the trauma profession [28]. These efforts alone might be insufficient but could be reinforced and scaled by collaboration with diverse civil society organizations, both locally and internationally, spanning spheres such as emergency care and response, injury prevention, infrastructure improvement, survivors’ groups for domestic or interpersonal violence, or veterans’ associations. A similar strategy of engaging a wide range of inter-related organizations has shown success in various cases from the Ottawa Process to Ban Land Mines to drafting the UN conventions on the Rights of the Child and Persons with Disabilities [29-31]. Therefore, advocates for improving the care for the injured might consider ways to catalyze network convergence, identify related issues (eg, resiliency of fragile states, road injury prevention and care, worker’s rights, child survival, economic productivity, social justice), and seek partners both in and beyond the health sector now that the importance of trauma care has been established [32,33]. Framing this platform in terms of a rights-based approach that cannot be overlooked if the global community seeks to achieve Sustainable Development Goal 3 “health for all” will be an important and compelling moral argument for action [34].

The DAH-TC donor and channel landscape is sparse compared to other global health foci; as a result, DAH-TC is particularly vulnerable to temporal economic trends and shifts in donor foreign or development policy. As an example, the 2008 global financial crisis significantly impacted DAH. In the two years preceding the crisis, DAH grew by 17% per year; DAH grew only 4% per year from 2009 to 2011 [35]. Further, funders responded differently to the crisis. The World Bank’s International Bank for Reconstruction and Development contributed substantially to DAH immediately after the crisis as a deliberate strategy in response to the recession to help developing countries stimulate their economies and provide safety nets for their citizens. Conversely, bilateral and United Nations Agencies contracted their DAH portfolios significantly, namely the result of reduced donations from member countries. Given that DAH-TC currently relies on bilateral and United Nations agencies as both donors and channels, any strain on monies available for DAH may have disproportionately negative effects on DAH-TC. To mitigate this risk, DAH-TC will need to diversify the landscape to include additional bilateral agencies and public-private partnerships, non-governmental organizations and foundations, which have been more resistant to financial crises [35].

Although war and collective violence are small contributors to the global burden of injury, 62% of DAH-TC flowed to recipients in conflict. This highlights the geopolitical drivers of DAH, and again demonstrates the importance of a diverse donor landscape to damper the effects of shifting geopolitical and developmental policy [36]. Certainly, countries embroiled in conflict incur disproportionate burdens of disease related both directly and indirectly to violence and need humanitarian relief immediately and development aid as they transition to post-conflict states [37,38]. However, the vast majority of the burden of injury occurs in LMICs with health systems more ready to effectively absorb DAH and rapidly commute aid into trauma care capacity improvements and benefit from the positive knock-on effects that might result from targeted health system strengthening initiatives (eg, pre-hospital emergency services, resuscitation capabilities, surgical capacity, rehabilitation education). Additionally, DAH-TC needs channels with expertise in trauma care to better identify potentially successful projects, direct strategic disbursement of funds, oversee accountability, improve the translation of objectives to action, and foster sustainability [39]. Channels with a focus on injury could also serve to advocate for DAH-TC and disburse monies over time to mitigate temporal economic trends and donor funding cycles.

Although this study provides an understanding of the DAH-TC landscape, several limitations merit discussion prior to interpreting the results. First, there may have been projects that indirectly result in improvements to trauma care that were not included due to our search criteria. However, we used a broad and widely inclusive list of keywords generated from landmark documents and guidelines in the realms of global health and trauma care. Given that the majority of the records retrieved by the search were excluded due to not being related to trauma care and due to the fact that the entire disbursement amount was counted for any tagged trauma care projects (not conventionally the case for IHME analyses – proportionate amounts are calculated according to frequency of keywords that appear), it is more likely that our search was over-inclusive [12]. Second, the records retrieved from the project-level databased of CRS, DAC and Development Bank databases may misrepresent the amount or type of DAH-TC, given that record descriptions were occasionally incomplete or vague. Resultantly, there may be some degree of bias in screening and project classification. However, this was mitigated to some extent by having two-independent reviewers and is likely inconsequential given the overall small proportion of DAH directed to trauma care. Third, there may be trauma care projects funded outside of OECD and other major actors that contribute to DAH-TC, such as donations from China. Although DAH from China is not known with any degree of accuracy, there is general consensus that China is becoming a significant actor in global health financing in many countries where injury burden is highest [40]. Lastly, given the crucial role that injury prevention efforts play in ameliorating the trauma burdens worldwide, further investigation to understand the degree of prevention-related DAH is warranted. For example, it is estimated that injury rates have declined most substantially where prevention efforts have been strongest, such as large reductions in road traffic injuries as a result of drunk driving advocacy and use of occupant restraints [4]. Despite these limitations, the findings from this study allow reasonable conclusions to be drawn about DAH-TC trends and shortfalls, and ways in which the global health and trauma care communities might advocate for strengthening care for the injured.

## CONCLUSION

DAH-TC is critically underfunded and receives a disproportionately low amount of funding compared to other global health foci with smaller burdens of disease. Additionally, the limited donor and channel landscape creates vulnerability with regard to economic trends and changing developmental policy. To improve trauma care in LMICs and the DAH-TC landscape, stakeholders might:

- Work with LMIC governments to mobilize domestic resources to bolster DAH-TC and foster national ownership of trauma care;
- Use advocacy more effectively by catalyzing network convergence, identifying related issues, and seeking partners both in and beyond the health sector; and
- Seek partners within the donor and channel communities to provide and direct more strategic DAH-TC disbursements.

## Additional material

Online Supplementary Document
