# Supplementary material for: Tracking global development assistance for trauma care: A call for advocacy and action
Source: J Glob Health. 2021 Mar 27;11:04007. doi: 10.7189/jogh.11.04007 (PMC8005307; doi:10.7189/jogh.11.04007)
Supplement: Online Supplementary Document [file jogh-11-04007-s001.pdf]

Appendix S1.

1. Keyword list for extracting relevant project-level data from OECD databases

**Keywords or phrases**		
acid throwing	first-aid	road transport
air medical transport	first-responder*	scald
ALS	flame*	scene control
ambulance*	fracture*	scene safety
amputee*	Glasgow Coma Scale	self harm
assault	gun*	self-harm
ATLS	head-injur*	shock
bite	helmet	skin graft*
blunt force	highway patrol	speed limit*
burn*	IMEESC	spine board
bystander*	injur*	spine immobilization
call management center	injury prevention	stab
casualt*	interpersonal violence	suicide
Centralised call processing	IPV	suicide prevention
Centralized call processing	landmine*	TBI
crash scene	lay person	terrorism
dispatch	Lay provider	toxic effect
drown*	lay provider*	traffic regulation*
ED	lay responder*	transport accident*
electrocution	layperson	trauma
electromyography	medical emergencies	trauma-related
emergency care	medical emergency	traumatic brain injur*
emergency department	mine*	traumatic fracture*
emergency medic*	neurotrauma	traumatology
emergency unit care	paramedic*	triage
EMS	poison*	UAN
EMT	post-injury	Universal access number
endotracheal intubation	pre-hospital	
firearm*	prehospital	
first aid	resuscitation	
first responder*	road traffic	

2. Summary of primary data sources

Selected Funding Entities	Source	Attributes & Search Strategy
OECD Countries	IHME DAH Database (via DAC & CRS OECD Project Reporting)	1990-2015; scanned project titles and descriptions to match trauma keyword list
World Bank	Online project database[1]	1990-2015; sector = 'health'; theme = 'injuries/NCDs' OR 'Natural disaster mgmt' OR 'HS performance'; status = 'closed' OR 'active'
Inter-American Development Bank (IDB)	Online project database[2]	1990-2015; sector = 'health'; status = 'closed' OR 'completed' OR 'approved' OR 'implementation'; scanned project titles and descriptions to match trauma keyword list
Asian Development Bank (ADB)	Online project database[3]	1990-2015; sector = 'health'; status = 'closed' OR 'active'; scanned project titles and descriptions to match trauma keyword list
African Development Bank (AfDB)	Online project database[4]	No health sector-specific projects in database
Pan American Health Organization (PAHO)	Executive Committee Documents[5]	1990-2015; searched for mention of injury or trauma care-related investments
The Bill and Melinda Gates Foundation (BMGF)	Annual Reports, Financial Statements[6]	1998-2015; searched for mention of injury or trauma care-related investments
Global Fund to Fight Aids, Tuberculosis and Malaria (GFATM)	Annual Reports[7]	2003-2015; searched for mention of injury or trauma care-related investments
Population Services International (PSI)	Annual Reports[8]	2010-2015; searched for mention of injury or trauma care-related investments
Catholic Relief Services (CRS)	Annual Reports	2001-2015, searched for mention of injury or trauma care-related investments
Management Sciences for Health	Annual Reports[9]	No reports available before 2014.

3. Categories for Trauma Care Project Disaggregation

[1] The World Bank. Projects & operations. Washington, DC: World Bank. http://www.worldbank.org/projects (accessed May 2015).

[2] Inter-American Development Bank. Online projects database. Washington, DC: IDB. http://www.iadb.org/projects/ (accessed May 2015)

[3] Asian Development Bank. Online project database. Manila: ADB. http://www.adb.org/projects/ (accessed May 2015).

[4] African Development Bank. Online project database. Tunis: AfDB. http://www.afdb.org/en/projects-andoperations/project-portfolio/ (accessed May 2015).

[5] Pan American Health Organization. Financial report and audited financial statement 2012. Washington, DC: PAHO. http://www.paho.org/hq/index.php?option=com_content&view=category&layout=blog&id=1258&Itemid=1160&la ng=en (accessed May 2015)

[6] Bill & Melinda Gates Foundation. Online Annual Reports. Seattle, WA: Bill & Melinda Gates Foundation. https://www.gatesfoundation.org/Who-We-Are/Resources-and-Media/Annual-Reports (accessed May 2015)

[7] Global Fund to Fight Aids, Tuberculosis and Malaria. Online Annual Reports. Geneva, Switzerland: GFATM. https://www.theglobalfund.org/en/archive/annual-financial-reports/

[8] Population Services International. Online Annual Reports. Washington, DC: PSI. https://www.psi.org/about/annual-reports/

[9] Management Sciences for Health. Online Annual Reports. Medford, MA: MSH. https://www.msh.org/about-msh/financial-data/annual-report

Disaggregation by Site	Disaggregation by Theme	Disaggregation by Intervention Topic
Prehospital	Service delivery	Burn/chemical
Emergency unit	HR/training	Mine
Operative/critical care	Leadership/governance	Poisoning/snakebite
Rehabilitation	Infrastructure	Domestic Violence
System-wide (safety, data, preparedness)	Information/research	Drown
		Road traffic injuries
		Self-harm
		War/conflict
		Trauma, general
